# Demonstrating the interplay between resilience, engagement, and wellbeing among Chinese teachers: the mediating role of self-efficacy

**DOI:** 10.3389/fpsyg.2026.1744771

**Published:** 2026-03-06

**Authors:** Jianbo Yang

**Affiliations:** School of Literature and Law, Zhengzhou Shengda University, Zhengzhou, Henan, China

**Keywords:** Chinese teachers, engagement, positive psychology, resilience, self-efficacy, well being

## Abstract

**Introduction:**

Teacher resilience has been widely recognized as an important psychological resource that supports educators' professional functioning and wellbeing. However, the mechanisms through which resilience influences teachers' work engagement and wellbeing, particularly the mediating role of self-efficacy, remain insufficiently explored in the Chinese educational context. This study aimed to examine resilience as a predictor of work engagement and wellbeing and to investigate the mediating role of self-efficacy.

**Methods:**

A total of 411 Chinese educators participated in the study. Data were collected through self-report questionnaires measuring resilience, self-efficacy, work engagement, and wellbeing. Structural equation modeling (SEM) was employed to test the proposed structural framework and mediation effects.

**Results:**

The results indicated that resilience significantly and positively predicted both teachers' work engagement and wellbeing. Resilience and self-efficacy jointly explained 43.6% of the variance in wellbeing. In addition, self-efficacy was found to be a significant mediator in the relationships between resilience and work engagement, as well as between resilience and wellbeing.

**Discussion:**

These findings underscore the roles of resilience and self-efficacy in enhancing teachers' engagement and psychological wellbeing. The study suggests that interventions aimed at strengthening resilience and efficacy beliefs may improve instructional effectiveness and support teachers' sustainable professional development in educational settings.

## 1 Introduction

In recent times, positive psychology (PP) has gained significant attention, focusing more on the positive facets of life and considering well being as a key factor in personal success (Derakhshan, 2022; Derakhshan and MacIntyre, 2026; Kim et al., 2018). The foundation of any educational institution lies in its educators, making it essential to prioritize their well being within the academic environment (Wang and Derakhshan, 2023). Indeed, teachers are regarded as crucial stakeholders within the educational system, as their role is significant in shaping their success (Darling-Hammond et al., 2020). Moreover, engagement is recognized as one of the fundamental components contributing to well being within the PERMA model (Seligman, 2018). This construct is characterized by a state of involvement and immersion in a task, commonly referred to as “flow” (Seligman, 2018). Teacher engagement is conceptualized as a psychological state marked by vigor and motivational drive toward one's professional responsibilities (Greenier et al., 2021). Although teachers' knowledge and skills are essential determinants of their effectiveness, empirical research suggests that their engagement is substantially affected by a range of affective features (Dirgantoro et al., 2024; Xiao et al., 2022). This indicates that educators' views, principles, and personality traits significantly influence their instructional efficacy and level of engagement (Bandura, 1977). Identifying the elements that influence teachers' well being and engagement, like resilience and self-efficacy, is crucial for formulating policies and practices that promote teacher development (Burić, 2019; Derakhshan et al., 2020; Han et al., 2021; Li et al., 2022; Wang X. et al., 2024). Recently, the concept of teacher resilience pertains to a person's aptitude to recover from adversities and to effectively adapt to fluctuating situations (Chu and Liu, 2022; Liu and Chu, 2022) by navigating challenges while maintaining their well being (Liu and Han, 2022; Mansfield et al., 2016). Resilient educators exhibit enhanced self-esteem and a heightened dedication to their professional responsibilities (Richards et al., 2016). Educators who demonstrate resilience possess a greater capacity to rebound from setbacks, thereby maintaining their enthusiasm and vigor over time. Research suggests that teachers exhibiting resilience are likely to maintain a more favorable perspective regarding their professional responsibilities and experience diminished levels of anxiety (Daniilidou et al., 2020).

Moreover, self-efficacy constitutes a critical determinant in evaluating a teacher's performance and well being (Liu et al., 2024; Ortan et al., 2021; Wang et al., 2025; Yüce et al., 2023; Zhi et al., 2024). According to social cognitive theory (SCT), teacher self-efficacy refers to an individual's self-assurance to effectively organize, plan, and implement the required actions to accomplish academic purposes (Han et al., 2020; Huang et al., 2019). Also, research has proven that teachers possessing elevated levels of self-efficacy are more inclined to maintain perseverance when confronted with challenges (Han and Wang, 2021; Hayes et al., 2020).

While resilience, engagement, and well being have all been extensively researched, the simultaneous exploration of their interrelationships—along with the mediating role of self-efficacy—remains relatively uncharted territory (Derakhshan et al., 2022, 2025; Liu et al., 2024; Wang et al., 2024). Nevertheless, this research has been mostly conducted in cross-cultural or Western settings, resulting in a confined comprehension of the way those concepts act in unique academic perspectives of China. The rapidly changing academic reforms, raising tensions among teachers and intense educational expectations, lead to a setting where mental resources like engagement, self-efficacy, and resilience can work distinctly in various settings. Therefore, a shift from overall theoretical views to certain realities of teachers in China is vital for creating a context-based comprehension of teachers' well being. Accordingly, this study is essential for advancing a sustainable and effective educational system that enhances the well being and engagement of educators, finally supporting the progress of the broader educational landscape in China.

By recognizing self-efficacy as a mediating variable, we can gain better visions of the mechanisms through which resilience impacts both well being and engagement. Resilience positively influences self-efficacy, which subsequently contributes to improved well being and increased engagement (Wang and Pan, 2023). Understanding that self-efficacy serves as a mediating factor facilitates the design of targeted intervention strategies. Educational institutions can develop targeted programs designed to enhance self-efficacy, which may subsequently bring about increased resilience and improvements in well being and student engagement (Mansfield et al., 2016). An appreciation of the substantial power of self-efficacy advocates for the allocation of resources and the provision of training programs that enhance both resilience and self-efficacy among educators.

## 2 Literature

### 2.1 Resilience

Resilience is a person's capacity to withstand and overcome the adversities of life (Wang Y. et al., 2024). In education, it refers to educators' capacity to navigate teaching difficulties and bounce back from adverse work experiences (Li and Lv, 2022). Teacher resilience encompasses not just bouncing back from difficulties or adverse teaching experiences, but also moving ahead and making progress (Mansfield et al., 2016). Indeed, navigating hard teaching situations frequently fosters professional development, allowing educators to thrive in their careers (Beltman, 2020; Gao et al., 2025). Resilience is understood as the ability that empowers individuals to effectively navigate stress, rebound from challenges, and maintain their dedication to work and well being (Han, 2022). (Derakhshan et al. 2022) highlighted that resilient educators should utilize coping techniques, like looking for help and setting purposes, to alleviate work-related stress and adverse feelings, ultimately promoting well being. Moreover, educators who possess greater resilience tend to experience joy and fulfillment more easily in their job (Proietti Ergün and Dewaele, 2021). Prior studies highlight the power of resilience in maintaining the well being of educators (Derakhshan et al., 2022; Han, 2022). They stated that educators who exhibit resilience possess enhanced capabilities in stress management, foster a positive outlook, and actively engage in reflective practices that contribute to their ongoing professional development (Capone et al., 2022). The results designate that resilience serves a dual function for educators: it facilitates their ability to navigate immediate challenges while simultaneously fostering a sense of fulfillment and promoting well being. In general, resilience is considered a basic mental resource that allows teachers to maintain efficiency, preserve their well being, and keep engagement in their career roles.

### 2.2 Well being

In the context of PP, well being is characterized as a beneficial attribute that allows individuals to flourish (Wang and Derakhshan, 2025; Wang et al., 2021). In essence, individuals with a positive outlook on life feel satisfied. Individuals with elevated well being tend to feel positive emotions, participate in constructive behaviors, and possess inclusive comprehension of their jobs (Proietti Ergün and Dewaele, 2021; Wang and Hemchua, 2022). The examination of well being embraces two primary aspects: the hedonic outlook prioritizes the quest for joy, whereas the eudaimonic viewpoint recognizes life's demands and highlights individual development by discovering determination, fostering relations, acquiring skills and agency, and accepting the difficulties and experiences that life presents. Collectively, these factors enhance a person's gratification (Wang et al., 2021). For educators, prioritizing the well being of both themselves and their students is essential for fostering robust relationships, cultivating positive classroom environments, and ultimately enhancing their performance (Wang and Derakhshan, 2023; Wang and Pan, 2023). The experience of well being among teachers both improves their success and increases their resilience in case of various stressors, like substantial workloads, challenging student behaviors, and leaders' burdens. This favorable emotional state functions as a shield, facilitating educators' ability to successfully deal with the challenges of their job and mitigating their burnout (Bentea, 2017). Generally, teachers' well being indicates a crucial element of professional maintainability, forming teachers' resilience, long-term career satisfaction, and teaching quality.

### 2.3 Work engagement

Engagement has emerged as a prominent concept and has garnered significant attention within the fields of PP over the past decade (Derakhshan and Taghizadeh, 2025; Zhang et al., 2025). This concept was initially conceptualized as a multidimensional concept reflecting the intellectual, emotional, and behavioral involvement of individuals in their career presentations (Han and Wang, 2021). It is fundamentally rooted in the theoretical framework of engagement itself, emphasizing the significance of eagerness and enjoyment as key motivators that enhance individuals' job performance (Han and Wang, 2021). It is also conceptualized as a heightened state of energy coupled with a profound identification with one's professional responsibilities that is described by three distinct dimensions: vigor, dedication, and absorption (Schaufeli et al., 2006). Vigor encompasses energy, commitment, and determination. Educators exhibit robust energy and a resilient mindset while on the job; they consistently strive to fulfill their responsibilities, demonstrating diligence and a commitment to do their best, and despite encountering challenges, they persevere and thrive (Li et al., 2023). Additionally, absorption encompasses an individual's deep engagement and focus on their tasks. Educators consistently maintain a strong focus on their tasks, approach their responsibilities with seriousness, often experience time slipping away during work hours, and often struggle to disconnect from their professional duties (Zhang et al., 2025). Dedication reflects attitudes toward work characterized by pride, engagement, and interest. Educators experience a sense of engagement in their roles, demonstrate enthusiasm in their tasks, and possess feelings of pride, motivation, and challenge (Schaufeli et al., 2006). Based on the literature, findings indicate that vigor and dedication are fundamental components of engagement, while absorption functions more as a result of this engagement (Kong, 2021). Therefore, working engagement remains as a main index of teachers' motivational power and professional devotion, which affects their well being, perseverance, and performance.

### 2.4 Self-efficacy

Self-efficacy is a central construct in understanding an individual's behaviors, cognitive processes, and reactions in the context of challenging and stressful circumstances (Helms-Lorenz and Maulana, 2016). Teacher efficacy is an educator's belief in their skills to effectively facilitate learning and manage classroom environments (Hoang and Wyatt, 2021). This construct plays a pivotal role in influencing instructional practices and, ultimately, student outcomes, and it refers to educators' self-perception regarding their ability to effectively plan and implement the activities required to gain instructive goals (Han and Wang, 2021). According to the SCT framework, an individual's decision-making processes in specific contexts are significantly influenced by their observations. The behaviors that are observed and subsequently retained in memory can influence future cognitive processes and social behaviors. It is posited that the interplay among cognitive and environmental factors significantly shapes an individual's actions. The former encompass elements such as knowledge, expectations, and attitudes, whereas the latter are associated with social norms, community accessibility, and the impact of interpersonal issues (Mahler et al., 2018). Recent research has investigated the associations between teachers' efficacy and a range of academic results, such as enhanced job satisfaction and reduced burnout (Burić and Moe, 2020; Helms-Lorenz and Maulana, 2016; Hoang and Wyatt, 2021). Furthermore, teacher efficacy has the potential to bolster their resilience, thereby enabling them to regulate the stresses and challenges of their profession with greater efficacy (Li, 2022; Wang and Pan, 2023). For example, self-efficacious educators tend to perceive challenges as opportunities for professional development rather than barriers (Wang Y. et al., 2024). Moreover, self-efficacy is related to well being, encompassing different dimensions of educators' lives (Derakhshan et al., 2022; Huang et al., 2019; Liang et al., 2022; Ortan et al., 2021; Zee and Koomen, 2016). Consequently, this predisposition can cause better achievement and fulfillment in their professional endeavors (Fathi et al., 2021). In general, self-efficacy acts as a vital mental system that forms teachers' well being.

### 2.5 Theoretical framework

The research primarily created a theoretical structure based on the Job Demands–Resources (JD-R) model to explain the hypothesized relationships among self-efficacy, well being, engagement, and resilience before using SEM. The JD-R model holds that individual sources like self-efficacy and resilience have a significant effect on forming people's motivational procedures and well being results. In this structure, resilience is conceptualized as an individual source that allows teachers to deal with job requirements, while self-efficacy acts as a mental system by which resilience leads to greater engagement and enhanced well being. SEM eases the investigation of indirect and direct impacts in these theoretically based structures, offering a comprehensive comprehension of intricated mental and motivational procedures. Thus, SEM was used to investigate the suggested JD-R–based model, enabling the estimation of the direct impact of resilience on well being and engagement simultaneously and the indirect impacts that are mediated by self-efficacy.

### 2.6 The present research

Given the vital function of well being in the success of educators (Fan and Wang, 2022; Zhang and Derakhshan, 2025), numerous researchers around the world have systematically investigated the factors that predict these psychological constructs. A thorough examination of the relevant literature reveals that the majority of prior research has investigated the interrelations among well being, engagement, resilience, and self-efficacy within the framework of language education (Han, 2022; Han and Wang, 2021; Heng and Chu, 2023; Kong, 2021; Wang X. et al., 2024; Wang and Pan, 2023; Xie, 2021). Besides, there exist some empirical studies investigating these variables within the context of general education (Brouskeli et al., 2018; Burić et al., 2019; Dirgantoro et al., 2024; Kutsyuruba et al., 2019; Reppa et al., 2023).

Brouskeli et al. (2018) inspected how resilience affects the well being of 201 teachers, revealing that resilience is a predictor of their well being. Likewise, Burić et al. (2019) investigated how resilience influences teacher well being. They administered two scales to a group of educators and discovered that resilience can boost their well being. A qualitative method was utilized in research conducted by Kutsyuruba et al. (2019) to investigate how teachers perceive the value of resilience in promoting their well being. Interviews revealed that the majority of participants considered resilience to be a vital component of their well being. Dirgantoro et al. (2024) explored how self-efficacy and resilience influence educator engagement, with personality traits acting as mediating factors. The research identified a notable connection between self-efficacy, resilience, and the engagement of teachers in their work, which is mediated by various personality traits. Han (2022) examined how resilience contributes to enhancing the mental health of 343 English teachers. The findings indicate that resilience can improve their mental well being. In a similar vein, Han and Wang (2021) scrutinized the correlation between self-efficacy among Chinese educators and their level of work involvement within educational contexts. The results proved that self-efficacious teachers are more engaged.

Moreover, Heng and Chu (2023) focused on a model that encompassed concepts such as self-efficacy, reflective practices, and resilience in their engagement in China. The study employed SEM and identified that all three constructs served as direct predictors of work engagement. Kong (2021) explored the relationship between educator efficacy and commitment, revealing that the involvement of Chinese educators is substantially affected by their well being and self-efficacy. The findings of the study indicate that those educators who possess great degrees of self-efficacy and well being are more inclined to demonstrate greater levels of engagement. Reppa et al. (2023) explored how educators' efficacy affects their well being. The research, including 258 teachers, indicated that self-efficacy is the strongest predictor of teachers' well being, especially in fostering student engagement. In a similar vein, Wang and Pan (2023) explored a model of work engagement in China, emphasizing the roles of teacher efficacy and resilience. Their research employed SEM and found that both self-efficacy and resilience are predictors of engagement, with self-efficacy demonstrating a stronger influence than resilience. Wang X. et al. (2024) scrutinized how teacher satisfaction and resilience influence the well being and self-efficacy of 375 teachers. The research showed that teaching satisfaction and resilience serve as mediators in the connection between self-efficacy and well being. Furthermore, resilience was recognized as essential for effectively managing stress, adapting positively, and regulating emotions. Also, Xie (2021) inspected the influence of resilience and emotional regulation on the engagement of Chinese instructors. The findings revealed a substantial correlation between teacher engagement and resilience.

Although the above-mentioned studies have considered the same variables of the study; However, the role of teachers' resilience in their well being and engagement in the general educational context still needs to be studied in China. Moreover, there has been a lack of investigations inspecting the simultaneous impact of resilience on both teachers' well being and their engagement. Additionally, earlier studies have failed to consider how teachers' efficacy mediates the connection between resilience, well being, and engagement. To fill this gap, this study seeks to explore the impact of resilience on the well being and engagement of teachers. Moreover, this research tried to evaluate the role of self-efficacy as a mediator in the association among these variables. Following these aims, three questions have been raised:

Q1: Are there significant associations among resilience, teacher self-efficacy, work engagement, and well being?
Q2: Does teacher self-efficacy mediate the relationship between resilience and well being?
Q3: Does teacher self-efficacy mediate the relationship between resilience and work engagement?

## 3 Method

### 3.1 Participants

The collected sample contained 455 responses. These responses were screened to make sure that there were no unengaged cases included. To do so, first, the patterns of answers were screened. This resulted in spotting 25 cases with constant patterns, 11 with increasing, and 2 with decreasing patterns. These patterns are a clear indication that the respondents did not take the questions seriously and filled them out with low engagement in the task. Moreover, as the questionnaires aimed to capture different attributes, it was presumed that the participants' answers should have a certain variability. Therefore, the standard deviations of each participant's answers to all items were calculated, and those who showed low variability in their answers (SD below 0.5) were also considered unengaged. This process resulted in excluding 44 cases (38 with odd patterns and 6 with low SDs), leaving the final clean sample with 411 cases. Their age fluctuated from 30 to 40 (*M* = 35.3, SD = 6.18).

### 3.2 Instrument

The first scale used in this study is the 10-item resilience scale developed by Campbell-Sills and Stein (2007), as a psychometrically refined short form of the original 25-item scale created by Connor and Davidson (2003). This scale functions as a comprehensive instrument for assessing resilience across multiple dimensions. The survey includes Likert-scale questions, where answers vary from 0 (not true at all) to 4 (almost always true). The next one is the teacher self-efficacy scale, which was developed by Tschannen-Moran and Hoy (2001). This questionnaire comprises 24 self-report items designed to assess teachers' perceptions regarding their competencies in implementing effective instructional strategies, engaging students, and managing classroom environments. Responses were assessed using a 5-point Likert scale, with values extending from 1 (“none”) to 5 (a great deal). The other is the engagement scale developed by Schaufeli et al. (2006), which was employed to assess a teacher's level of job engagement. Each item was evaluated on a seven-point scale, from 0 (never) to 6 (always). This scale evaluates three dimensions: vigor and absorption, each with six items, and dedication, which has five items. The last is the 16-item scale designed by Collie et al. (2015) to evaluate well being across three fundamental dimensions. The first emphasizes professional well being, considering the various challenges associated with an individual's occupational roles. The second, organizational well being, significantly shapes educators' perceptions of the school as a multifaceted entity, encompassing various aspects such as administrative management, institutional culture, and educational practices. The third is concerned with the quality of learner interaction, encompassing the rapport between educators and learners, the comprehension of student behavior, and the implementation of motivational strategies. Each item is assessed using a 7-point Likert scale, which spans from negative to positive responses.

### 3.3 Data collection

The data were gathered through scales administered via an online platform known as “Questionnaire Star” during June and July 2023. Initially, the scholar validated, culturally adapted, and translated the questionnaire to make sure that its aspects precisely measure well being in the academic settings of China. Within those processes, all four tools were systematically adjusted and validated in the present research to make sure the measurement reliability, conceptual consistency, and cultural relevance for the teachers in China. Then, the scales were sent to college instructors from diverse regions throughout China who consented to participate voluntarily. They were subsequently requested to distribute the questionnaire to their coworkers through email. The questionnaires were used to gather data concerning teacher self-efficacy, resilience, engagement, and well being, and they needed to allocate approximately 40 min to complete them. Before participating in the study, educators were provided with a comprehensive briefing regarding the objectives of the research, and they were assured regarding the confidentiality of their responses. They were also informed of their right to withdraw at any point without any problems. All procedures performed in studies involving human participants were in accordance with the ethical standards of the 1964 Helsinki declaration and its later amendments. It was approved by the institutional review board of Zhengzhou Shengda University.

### 3.4 Data analysis

The reliability of each tool was assessed, and the associations between the variables were validated by examining the observed covariance within the model. As the theory behind the study, i.e., JD-R model, emphasizes on the complex effects of job demands on outcomes (Granziera et al., 2021; Skaalvik and Skaalvik, 2018), structural equation modeling was selected to capture the possible complex connections among the variables. SEM allows for the simultaneous analysis of multiple dependent and independent variables, enabling researchers to empirically test theoretical models. Unlike traditional statistical methods, it offers several benefits, such as the ability to analyze latent variables and account for measurement errors. Additionally, SEM facilitates the examination of both direct and indirect effects within a proposed framework, providing a thorough understanding of complex phenomena. This study employed SEM to explore the interactions among the variables.

## 4 Results

A confirmatory factor analysis (CFA) model was run to both measure the loadings of the items and make sure that the questionnaires were valid in the given context. Also, the convergent validity of the constructs was inspected through inspection of the standardized and unstandardized loadings (Table 1).

**Table 1:** Estimated loadings in the CFA model.

**Observed Variable**	**Path**	**Latent variable**	Unstandardized				**Standardized**
			**Estimate**	**S.E**.	**C.R**.	***P***	**Estimate**
Dedication	< –	TWE	1.000				0.533
Absorption	< –	TWE	0.899	0.134	6.710	0.000	0.538
Vigor	< –	TWE	1.513	0.185	8.185	0.000	0.989
EIS	< –	Efficacy	1.000				0.967
ESE	< –	Efficacy	0.995	0.122	8.168	0.000	0.875
ECM	< –	Efficacy	1.009	0.119	8.498	0.000	0.906
Workload	< –	Well being	1.000				0.986
S. Interaction	< –	Well being	0.990	0.039	25.321	0.000	0.986
Organizational	< –	Well being	0.939	0.039	23.835	0.000	0.985
R01	< –	Resilience	1.000				0.848
R02	< –	Resilience	0.981	0.044	22.476	0.000	0.850
R03	< –	Resilience	0.996	0.043	23.371	0.000	0.868
R04	< –	Resilience	0.983	0.043	23.005	0.000	0.861
R05	< –	Resilience	1.006	0.044	22.864	0.000	0.858
R06	< –	Resilience	0.954	0.044	21.681	0.000	0.832
R07	< –	Resilience	1.026	0.045	22.682	0.000	0.854
R08	< –	Resilience	1.019	0.044	23.041	0.000	0.861
R09	< –	Resilience	1.005	0.044	23.072	0.000	0.862
R10	< –	Resilience	0.987	0.043	23.118	0.000	0.863
VI01	< –	Vigor	1.000				0.650
VI02	< –	Vigor	0.973	0.097	10.013	0.000	0.621
VI03	< –	Vigor	0.638	0.083	7.716	0.000	0.453
VI04	< –	Vigor	1.014	0.101	10.054	0.000	0.624
VI05	< –	Vigor	0.591	0.081	7.270	0.000	0.424
VI06	< –	Vigor	0.677	0.081	8.313	0.000	0.494
DE01	< –	Dedication	1.000				0.805
DE02	< –	Dedication	1.033	0.049	21.046	0.000	0.859
DE03	< –	Dedication	0.536	0.051	10.423	0.000	0.492
DE04	< –	Dedication	0.607	0.058	10.532	0.000	0.497
DE05	< –	Dedication	1.160	0.048	24.312	0.000	0.984
AB01	< –	Absorption	1.000				0.698
AB02	< –	Absorption	0.750	0.068	11.086	0.000	0.594
AB03	< –	Absorption	0.643	0.072	8.889	0.000	0.473
AB04	< –	Absorption	0.931	0.080	11.645	0.000	0.626
AB05	< –	Absorption	0.753	0.071	10.546	0.000	0.564
AB06	< –	Absorption	1.180	0.077	15.385	0.000	0.906
WL01	< –	Workload	1.000				0.877
WL02	< –	Workload	0.951	0.037	25.515	0.000	0.872
WL03	< –	Workload	0.908	0.037	24.432	0.000	0.855
WL04	< –	Workload	0.962	0.037	26.035	0.000	0.881
WL05	< –	Workload	0.928	0.036	25.506	0.000	0.872
WL06	< –	Workload	0.947	0.038	24.883	0.000	0.862
OR01	< –	Organizational	1.000				0.870
OR02	< –	Organizational	0.992	0.039	25.252	0.000	0.874
OR03	< –	Organizational	1.047	0.043	24.549	0.000	0.862
OR04	< –	Organizational	1.040	0.041	25.298	0.000	0.875
OR05	< –	Organizational	0.981	0.040	24.708	0.000	0.865
OR06	< –	Organizational	1.028	0.040	25.491	0.000	0.878
SI01	< –	S. Interaction	1.000				0.896
SI02	< –	S. Interaction	0.954	0.035	27.017	0.000	0.879
SI03	< –	S. Interaction	0.997	0.038	26.207	0.000	0.868
SI04	< –	S. Interaction	0.938	0.037	25.674	0.000	0.860
SE01	< –	EIS	1.000				0.545
SE02	< –	EIS	1.202	0.128	9.373	0.000	0.605
SE03	< –	EIS	1.194	0.123	9.699	0.000	0.637
SE04	< –	EIS	1.303	0.128	10.139	0.000	0.685
SE05	< –	EIS	1.206	0.127	9.488	0.000	0.616
SE06	< –	EIS	1.052	0.124	8.491	0.000	0.524
SE07	< –	EIS	1.327	0.127	10.447	0.000	0.722
SE08	< –	EIS	0.847	0.113	7.512	0.000	0.445
SE09	< –	ECM	1.000				0.568
SE10	< –	ECM	1.201	0.117	10.281	0.000	0.662
SE11	< –	ECM	1.169	0.115	10.130	0.000	0.647
SE12	< –	ECM	1.258	0.114	11.016	0.000	0.738
SE13	< –	ECM	1.101	0.111	9.946	0.000	0.630
SE14	< –	ECM	1.035	0.112	9.235	0.000	0.567
SE15	< –	ECM	1.182	0.117	10.078	0.000	0.642
SE16	< –	ECM	1.206	0.118	10.243	0.000	0.658
SE17	< –	ESE	1.000				0.546
SE18	< –	ESE	1.090	0.121	9.033	0.000	0.588
SE19	< –	ESE	1.215	0.122	9.919	0.000	0.683
SE20	< –	ESE	0.822	0.116	7.076	0.000	0.421
SE21	< –	ESE	1.186	0.117	10.162	0.000	0.713
SE22	< –	ESE	0.979	0.113	8.660	0.000	0.553
SE23	< –	ESE	1.045	0.120	8.718	0.000	0.558
SE24	< –	ESE	0.984	0.113	8.704	0.000	0.557

As reported in Table 1, all items had significant loadings on their corresponding components. Moreover, while all components had acceptable standardized loadings to their corresponding constructs, two items showed slightly low loadings (items highlighted in the table). Given that the components' loadings were safely high, only the two items with loadings below 0.45 (item 5 from vigor and item 8 from efficacy in instructional strategies) were dismissed. Table 2, below reported the observed values alongside the thresholds of different model fit indices.

**Table 2:** CFA model's goodness of fit.

**Criteria**	**Observed values**	Thresholds			
		**Poor**	**Acceptable**	**Excellent**	**Evaluation**
CMIN	3,935.47				
DF	1,999				
CMIN/DF	1.969	>5	< 5	>3	Excellent
RMSEA	0.049	>0.08	< 0.08	< 0.06	Excellent
CFI	0.916	< 0.9	>0.9	>0.95	Acceptable
TLI	0.914	< 0.9	>0.9	>0.95	Acceptable
SRMR	0.042	>0.10	>0.08	< 0.08	Excellent

**Figure 1:**
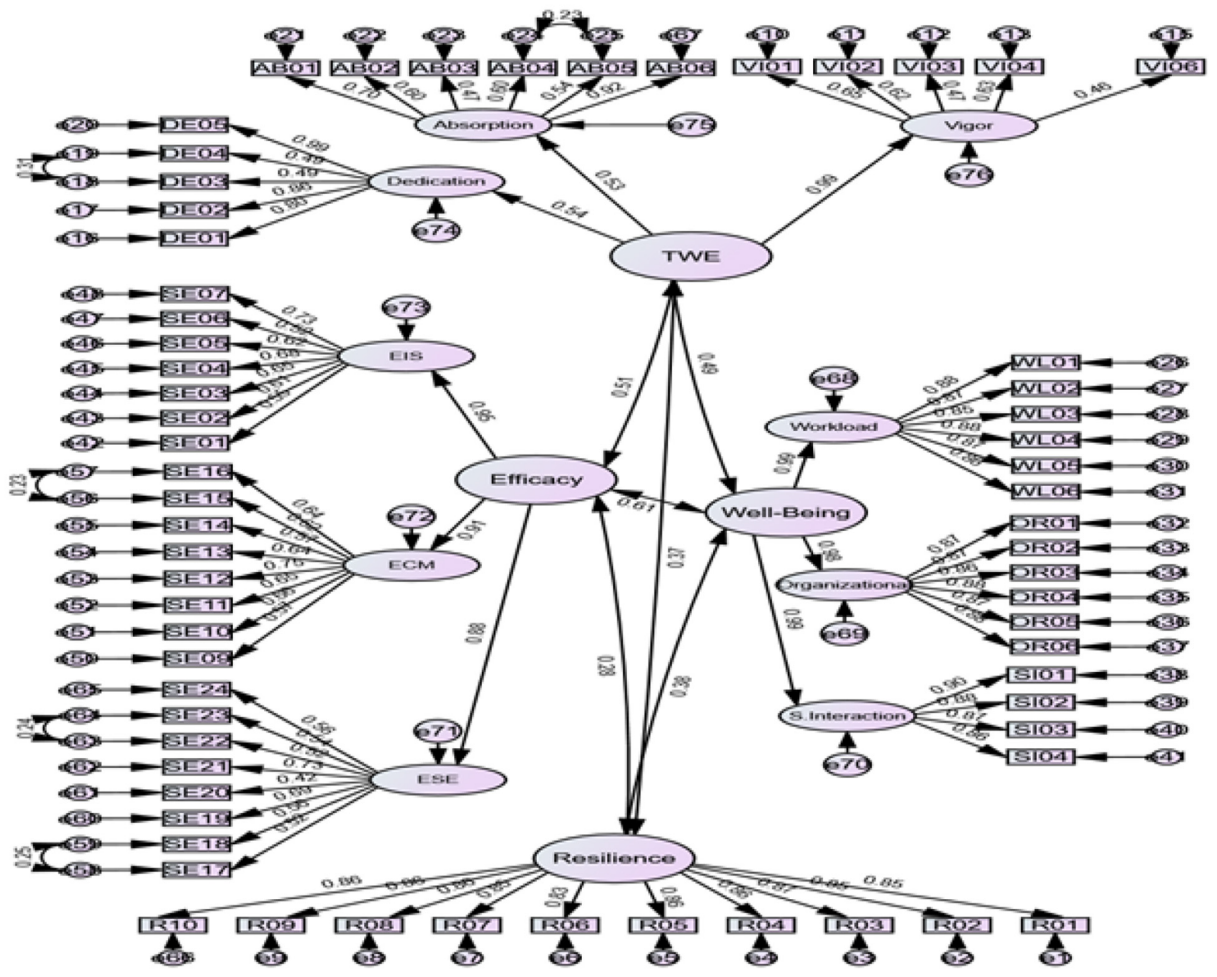
CFA model with standardized estimates.

As reported in Table 2, the model demonstrated acceptable to excellent fit indices. The chi-square test was significant, and the degree of CMIN/df fell within the excellent range of 1–3. The mean root square of error approximation (RMSEA), which is an absolute index that measures how different the observed model is from the perfect model, was 0.049, indicating the low variance between the two models. The comparative fit index (CFI) and Tucker-Lewis index (TLI) were above 0.91, indicating that the discrepancy between the data and hypothesized model was less than 0.09. Finally, the standardized root mean square residual (SRMR) implied correlation matrix. The low values are indicative of the low variance between the two models. The observed value of 0.042 also shows an excellent fit. The final model is presented in Figure 1.

As reported in Table 3, above, the composite reliability (CR) values for all four constructs exceeded the critical value of 0.7 and the high values of MaxR(H) confirm the internal consistency. The average variance explained (AVE) in each case was safely above 0.5 and the maximum shared variance (MSV) was lower than AVE. These conditions support the convergent validity of the model. Finally, for all four constructs, the square root of AVE was above their associations with other constructs. The above Table also answers the first research questions of the study. The results showed that all four variables of the study have significant inter-correlation with one another at *p* < 0.01. Resilience had positive and moderate correlations with efficacy (*r* = 0.376), engagement (*r* = 0.370), and well being (*r* = 0.281). The correlations between well being and engagement (*r* = 0.489), well being and efficacy (*r* = 0.606), as well as engagement and efficacy (*r* = 0.514) were also positive and strong.

**Table 3:** Reliability and validity.

					Fornell–Larcker criterion			
**Constructs**	**CR**	**AVE**	**MSV**	**MaxR(H)**	**Resilience**	**Well being**	**TWE**	**Efficacy**
Resilience	0.965	0.732	0.141	0.965	**0.856**			
Well being	0.990	0.972	0.367	0.990	0.376^**^	**0.986**		
TWE	0.743	0.514	0.264	0.978	0.370^**^	0.489^**^	**0.717**	
Efficacy	0.939	0.837	0.367	0.947	0.281^**^	0.606^**^	0.514^**^	**0.915**

^**^Correlation is significant at *p* < 0.01.

**Figure 2:**
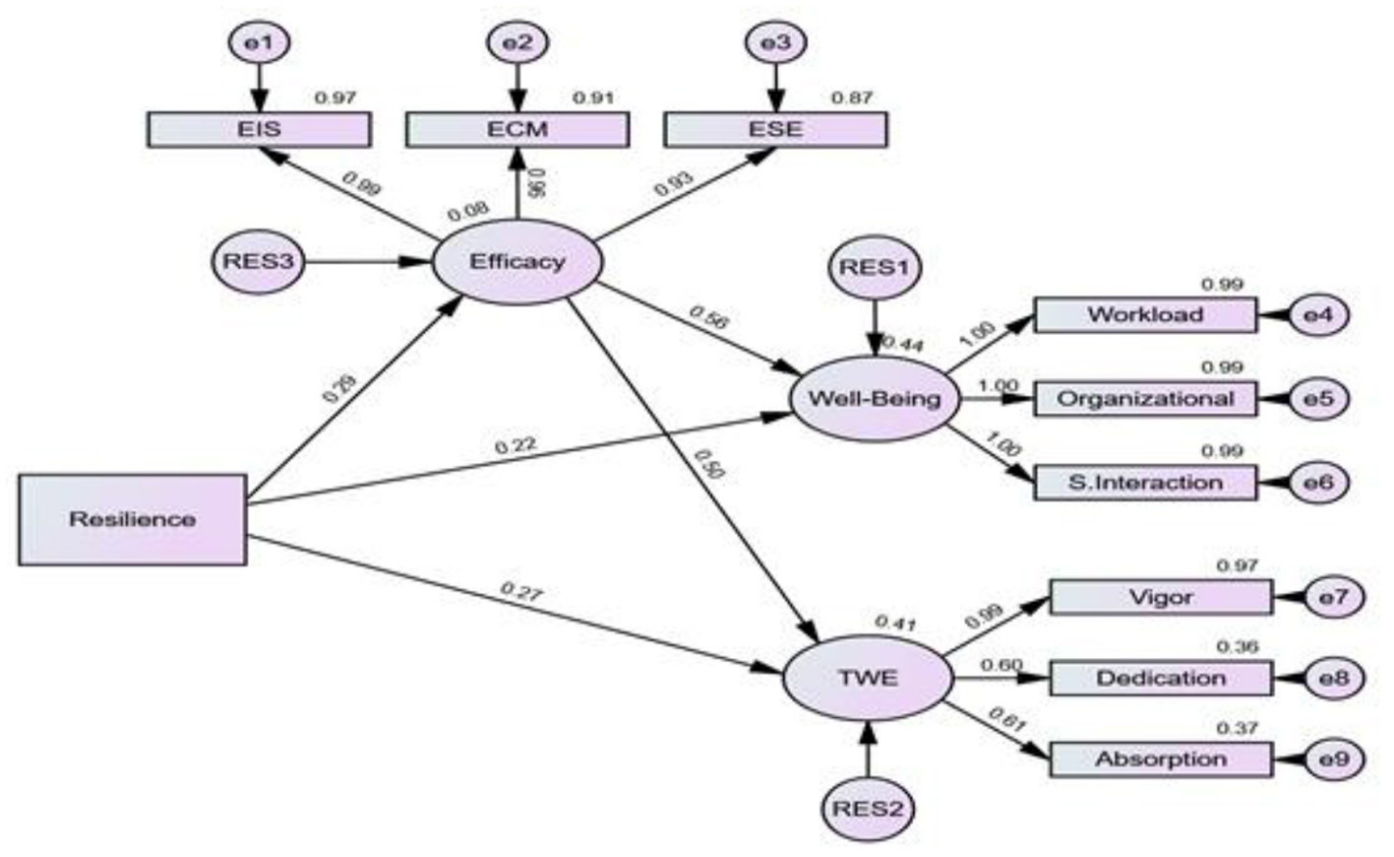
Regression model with standardized estimates.

To answer the second and third research questions, a measurement model using SEM was created. To reach weighted factors, first, the data in the CFA model, above, was, imputed using regression imputation, and the values obtained were used in the measurement model. The model is depicted in Figure 2.

The results in Table 4 and Figure 2 indicate that resilience and efficacy are both predictors of well being, jointly explaining 43.6% of its variance. They were significant predictors of engagement, jointly explaining 40.7% of its variance. Resilience could uniquely explain 8.5% of the change in teacher efficacy. The inspection of the mediating effects (indirect effects) showed significant results (*p* < 0.05), indicating that efficacy is a significant mediator in the associations between both resilience and well being as well as resilience and engagement.

**Table 4:** The measurement model.

**Outcome variable**	**Path**	**Predictor variable**	Unstandardized				**Standardized estimate**	**Bootstrap**		
			**Estimate**	**S.E**.	**C.R**.	**P**		**Lower**	**Upper**	***P***
Efficacy	< –	Resilience	0.157	0.026	6.090	0.000	0.291			
Well being	< –	Resilience	0.210	0.037	5.666	0.000	0.222			
Well being	< –	Efficacy	0.988	0.070	14.132	0.000	0.561			
TWE	< –	Resilience	0.257	0.039	6.663	0.000	0.272			
TWE	< –	Efficacy	0.883	0.073	12.160	0.000	0.504			
Well being	< –	Indirect effect	0.155				0.163	0.104	0.217	0.010
TWE	< –	Indirect effect	0.138				0.147	0.097	0.204	0.010

## 5 Discussion

Based on the findings, it was revealed that teacher resilience was positively linked to well being. Indeed, resilience facilitates educators in sustaining a constructive perspective and persevering in the face of challenges, thereby enhancing their well being (Derakhshan et al., 2022; Zhi and Derakhshan, 2024). This finding aligns with Wang et al.'s (2024) research, which emphasized that resilient educators possess enhanced capabilities to manage stress effectively and sustain their well being. It mitigates the sense of helplessness while enhancing their achievement and satisfaction, consequently contributing to better well being. It is posited that instructors demonstrating greater resilience are more adept at effectively managing the environments of the educational institutions in which they operate, consequently experiencing lower levels of anxiety. This result is along with the one conducted by Wang and Pan (2023), which identified a significant correlation between teacher well being and resilience.

Moreover, teacher resilience was positively linked to engagement. The research reveals that teacher resilience serves as a significant determinant of the work engagement levels among EFL instructors. The findings underscore the significance of resilience in the teaching profession, indicating that educators who embody resilience are more adept at navigating challenging circumstances and alleviating adverse consequences. In essence, elevated levels of resilience facilitate teachers' ability to collaborate more effectively with colleagues and enhance their job satisfaction. This sense of satisfaction, in turn, serves as a facilitator for increased engagement in their instructional roles (Mansfield et al., 2016). These findings corroborate the conclusions put forth by Brouskeli et al. (2018), who determined that teacher resilience serves as a significant predictor of their well being. In a similar vein, Burić et al. (2019) reported that resilience exerts a positive impact on teachers' well being, underscoring its protective function against adverse emotional states, burnout, and psychopathological symptoms.

Besides, it was revealed that teacher resilience was linked to their efficacy. Furthermore, self-efficacy enhances teachers' confidence in their capabilities and promotes resilience (Li, 2022). When educators exhibit elevated levels of self-efficacy, they demonstrate robust confidence in their capacity to address and navigate challenges that arise within the classroom environment (Capone et al., 2022). This sense of confidence fosters resilience, thereby enabling individuals to effectively manage stress and mitigate the risk of burnout (Wang et al., 2024). The results align with those of Daniilidou et al. (2020) and Yada et al. (2021), suggesting that self-efficacy serves as a basis for diminishing apprehension, while also fostering resilience. Resilient educators can excel in challenging circumstances, find fulfillment in their roles, control their emotions, navigate situations efficiently, deliver effective instruction, and remain committed to their teaching careers (Hu, 2023; Wang and Pan, 2023).

Furthermore, the results suggest that enhancing self-efficacy may positively impact teachers' engagement. The findings support those of empirical research indicating that a robust sense of self-efficacy contributes to enhanced teacher engagement, thereby also fostering a more positive learning climate (Heng and Chu, 2023). The findings echo the study by Buric and Macuka (2018), who suggested that self-efficacious educators demonstrate increased energy and a stronger commitment to their professional duties, and the affirmative relationship between self-efficacy and engagement is attributable to the confidence exhibited by individuals with high self-efficacy in the likelihood of achieving positive outcomes. This finding is in agreement with the work of Granziera and Perera (2019), Han and Wang (2021), and Wang and Pan (2023), who showed that self-efficacy serves as a positive predictor of teachers' engagement. In a general context, individuals possessing elevated self-efficacy beliefs are inclined to exhibit superior performance in their professional endeavors. The explanation may be rooted in Bandura's (2010) SCT. Teachers who possess confidence in their instructional expertise are likely to experience increased intrinsic motivation.

Moreover, regarding the mediating role of self-efficacy, it can be stated that self-efficacious educators are more prone to engage in their work, thereby cultivating a supportive atmosphere that contributes to the enhancement of their well being. These educators possess confidence in their capacity to address challenges within the classroom environment, thereby cultivating resilience. This resilience facilitates effective stress management and diminishes the likelihood of burnout, thereby contributing to an enhancement of well being. This is congruent with the outcomes of Hu's (2023) work, indicating that teachers exhibiting resilience possess enhanced capabilities in managing their emotions, a factor that is essential for maintaining their well being. This result is consistent with prior research (Huang et al., 2019; Xiao et al., 2022), proving that educators possessing high self-efficacy tend to experience enhanced well being, increased commitment to their teaching responsibilities, and a reduction in stress and burnout. This aligns with Zee and Koomen (2016) that self-efficacy serves as a fundamental determinant of well being and plays a significant role in shaping both personal and professional trajectories. Furthermore, research indicates that educators who perceive a sense of control over their professional activities, along with possessing the requisite skills and resources to effectively tackle challenges, exhibit a diminished likelihood of experiencing stress and burnout (Leijen et al., 2024). The ability to effectively manage stressors and navigate challenging circumstances is positively correlated with enhanced well being.

Regarding the theory of the study, the JD-R model provides a theoretical framework for this study, suggesting that job resources may alleviate the adverse effects of job demands on work-related outcomes (Granziera et al., 2021; Skaalvik and Skaalvik, 2018). In this framework, self-efficacy is conceptualized as an intrinsic resource that empowers educators to navigate job demands with greater efficacy, thereby contributing to enhanced well being and increased levels of engagement. Educators who possess strong self-efficacy can tackle challenges and deal with difficulties, enhance their resilience, and enable them to manage job demands more effectively. Teachers who demonstrate resilience and possess a strong sense of self-efficacy tend to enjoy more positive emotions and reduced stress, thereby improving their well being. Moreover, high self-efficacy cultivates increased enthusiasm and commitment to one's work.

## 6 Conclusion

The findings from the SEM analyses reveal a significant outcome: the resilience of Chinese teachers plays a crucial role in enhancing their engagement and well being. Moreover, it can be reasonably concluded that the factors influencing teachers' engagement and well being extend beyond just their personal resources. In addition, other elements like self-efficacy can significantly influence teachers' involvement and well being. Recognizing self-efficacy as a mediating factor reveals that resilience contributes to a stronger sense of self-efficacy, subsequently improving both well being and engagement. This insight offers a more detailed perspective on the ways in which resilience influences these results. This research indicates that resilience improves well being and engagement through increased self-efficacy. This suggests that teachers' sense of confidence in their teaching skills. In essence, educators exhibiting greater resilience may possess enhanced capabilities to effectively manage the stressors inherent in their profession, including elevated workloads, challenging student behaviors, and demanding classroom conditions. The capacity to effectively manage stressors enables instructors to preserve a sense of well being. Enhanced resilience can foster greater teacher self-efficacy, which helps mitigate stress, thereby boosting their engagement.

Educators exhibiting high levels of self-efficacy are more inclined to adopt innovative pedagogical practices, demonstrate enhanced classroom management abilities, and actively participate in professional development opportunities compared to their counterparts with low self-efficacy. High efficacy pertains to an individual's confidence in their capacity to execute tasks and attain objectives, a construct that is associated with better outcomes. The findings present that educational institutions should prioritize the enhancement of self-efficacy among Chinese educators, as such improvements are correlated with more effective pedagogical behaviors, including heightened engagement, and the promotion of resilience.

This study further contributes to the existing body of literature by emphasizing the significance of particular teacher attributes, notably self-efficacy, which can be strategically addressed in interventions and programs designed to enhance teacher engagement and well being. In addition, the research underscores the necessity of formulating interventions that explicitly address teacher self-efficacy and resilience to enhance their well being and engagement. Interventions aimed at enhancing these characteristics may encompass a range of initiatives, including structured training and development programs, mentoring and coaching frameworks, as well as mindfulness and stress reduction interventions. Consequently, it is recommended that trainers, teacher educators, institutional administrators, curriculum developers, and education policymakers prioritize the enhancement of teacher self-efficacy within their workshops. Educational institutions may implement workshops and training sessions aimed at fostering resilience and enhancing self-efficacy among students, which can help build confidence and enhance classroom management skills. Institutions can use resilience-development workshops like mindfulness-oriented plans, which assist teachers in lowering stress and adjusting emotions. These programs frequently incorporate techniques for stress management as well as exercises designed to enhance resilience. Indeed, the integration of resilience-building strategies within teacher training programs represents a proactive approach that has the potential to substantially improve their well being and engagement. By providing teachers with resources and strategies to effectively manage stress and adapt to challenges, educational institutions can promote a more stable and contented teaching workforce. This phenomenon consequently enhances the broader educational community by facilitating the establishment of a more supportive and effective learning environment. Moreover, it is recommended that more studies be conducted from the existential positive psychology (EPP; Derakhshan and MacIntyre, 2025a,b, 2026; Derakhshan and Park, 2026), where both positive and negative emotions should be investigated.

This study presents several limitations that must be acknowledged when interpreting the findings. Initially, the study employed self-report measures so future research could benefit from the incorporation of objective measures of teacher well being, including observation. Such approaches would enhance the validity of findings by complementing self-report data, thereby offering a more inclusive view of the variables of the study. Secondly, longitudinal designs are essential for the determination of causal relationships among variables. Furthermore, experimental designs may be utilized to evaluate the efficacy of interventions designed to improve teacher self-efficacy, resilience, and engagement. These methodologies can also facilitate the investigation of the mechanisms by which such treatment affects teacher well being.

## Data Availability

The raw data supporting the conclusions of this article will be made available by the authors, without undue reservation.
